# Remote actuation and on-demand activation of biomaterials pre-incorporated with physical cues for bone repair

**DOI:** 10.7150/thno.97610

**Published:** 2024-07-16

**Authors:** Xueping Kong, Tianyi Zheng, Zhaoyi Wang, Tong Zhou, Jiezhong Shi, Ying Wang, Ben Zhang

**Affiliations:** Sinopec Key Laboratory of Research and Application of Medical and Hygienic Materials Sinopec (Beijing) Research Institute of Chemical Industry Co., Ltd., 14 Beisanhuan East Road, Chao Yang District, Beijing 100013, China.

**Keywords:** biomaterials, bone repair, on-demand activation, physical cues, remote actuation

## Abstract

The high incidence of bone defect-related diseases caused by trauma, infection, and tumor resection has greatly stimulated research in the field of bone regeneration. Generally, bone healing is a long and complicated process wherein manipulating the biological activity of interventional scaffolds to support long-term bone regeneration is significant for treating bone-related diseases. It has been reported that some physical cues can act as growth factor substitutes to promote osteogenesis through continuous activation of endogenous signaling pathways. This review focuses on the latest progress in bone repair by remote actuation and on-demand activation of biomaterials pre-incorporated with physical cues (heat, electricity, and magnetism). As an alternative method to treat bone defects, physical cues show many advantages, including effectiveness, noninvasiveness, and remote manipulation. First, we introduce the impact of different physical cues on bone repair and potential internal regulatory mechanisms. Subsequently, biomaterials that mediate various physical cues in bone repair and their respective characteristics are summarized. Additionally, challenges are discussed, aiming to provide new insights and suggestions for developing intelligent biomaterials to treat bone defects and promote clinical translation.

## Introduction

In recent decades, a large number of patients have suffered bone defects caused by bone tumors, osteoporosis, arthritis, and other related diseases, requiring orthopedic repair materials. Bone repair is a complicated, precise, and slow process that can be tuned by various factors. In addition to basic structural support, bone repairing materials are also desired to meet the specific functionalities performed by bioactive factors involved in bone healing at different repair stages. Accordingly, various synthetic scaffolds have been developed to provide biocompatible and bioactive platforms for bone tissue regeneration 1-4. Physical cues such as heat, electricity, magnetism, ultrasound, light, and mechanical forces provide superior convenience with remote operation and have been extensively studied for their safety and efficacy in promoting bone regeneration by activating endogenous regulators 5. However, how to well integrate physical cues with biomaterials to achieve precise and controlled modulation is challenging. This reveiw will discuss these physical cues pre-integrated into bone-repairing materials and activated by exogenous stimuli with high biosafety and strong tissue penetration, such as light, electricity, and magnetic field, enabling remote actuation and on-demand activation for bone regeneration.

As an emerging treatment modality, thermal-based therapy has made remarkable progresses in the treatment of tumors 6, osteoarthritis 7, bone infection 8, and other bone-related diseases 9. Studies have shown that a mild thermal effect (40-43 ℃) favors cell activity, proliferation and differentiation, thereby promoting bone defect repair. The highest osteogenic activity is observed with thermal stimulation at 42 °C twice a day, as prolonged exposure times at a certain temperature result in tissue anesthesia and cell death, whereas short exposure times have no effect 10,11. However, the outcomes may be different for different disease models and laser parameters, and extensive trials are needed to determine the optimal experimental conditions. In order to increase the utilization of thermal effect and meanwhile enable the safety of surrounding healthy tissues, exogenous stimuli that penetrate deep into the tissues, such as near-infrared (NIR) light 12 and magnetic field 13, are often used to activate implanted biomaterials *in situ* and generate local thermal energy for treatment. Accordingly, photosensitizers with strong NIR light absorption and high photothermal conversion efficiency have been developed for bone defect repair 14. Besides light-induced thermal effect, resonance between the spin magnetic moment of the magnetic composite scaffold and an external alternating magnetic field (AMF) can also provide a local warm environment to promote bone repair 15.

Bioelectricity is an important physiological activity of living organisms, and bone is a natural piezoelectric material; therefore, electrical signals can be used as stimuli to promote bone growth and remodeling. Electrical stimulation can regulate many biological processes, ranging from cell cycle, migration, proliferation, restoring nerve conduction, muscle contraction, embryogenesis, and tissue regeneration 16. Restoration of the natural bioelectric properties of healthy bone tissue using electroactive biomaterials with electrode-free, wireless, and self-charging capabilities, such as piezoelectricity, provides a favorable microenvironment for bone regeneration 17. Commonly used electroactive materials in the biomedical field mainly include conductive materials (such as carbon-based nanomaterials, metallic materials, and conductive polymers, etc.), piezoelectric materials (like piezoelectric ceramics, piezoelectric polymers, etc.) 18 and other electroactive materials (magnetoelectric and photoelectric materials, etc.).

Low-frequency (below 1 MHz) homogeneous magnetic fields have received much attention for their ability to penetrate living tissues 19. In addition, magnetic fields can improve blood circulation at bone defects, stimulate osteoblast and chondrocyte generation, inhibit the activity of osteoclasts, and accelerate bone calcification, which helps combat osteoporosis and bone defects 20-22. Magnetic nanoparticles (MNPs) have been widely used in drug delivery, magnetic resonance imaging, and bone repair due to their excellent biocompatibility and magnetism 23,24. Under an exogenous magnetic field, the MNPs are rapidly magnetized, exerting a sustained weak magnetic force on the cells, thus making the superparamagnetic scaffolds better for bone regeneration than the magnetic field or MNPs alone. The positive effect of magnetic scaffolds, remotely coupled with magnetic fields, was confirmed on cell differentiation 25. Although the exact mechanism is still being investigated, this non-/less invasive bone repair strategy is still of great interest.

**Figure 1:**
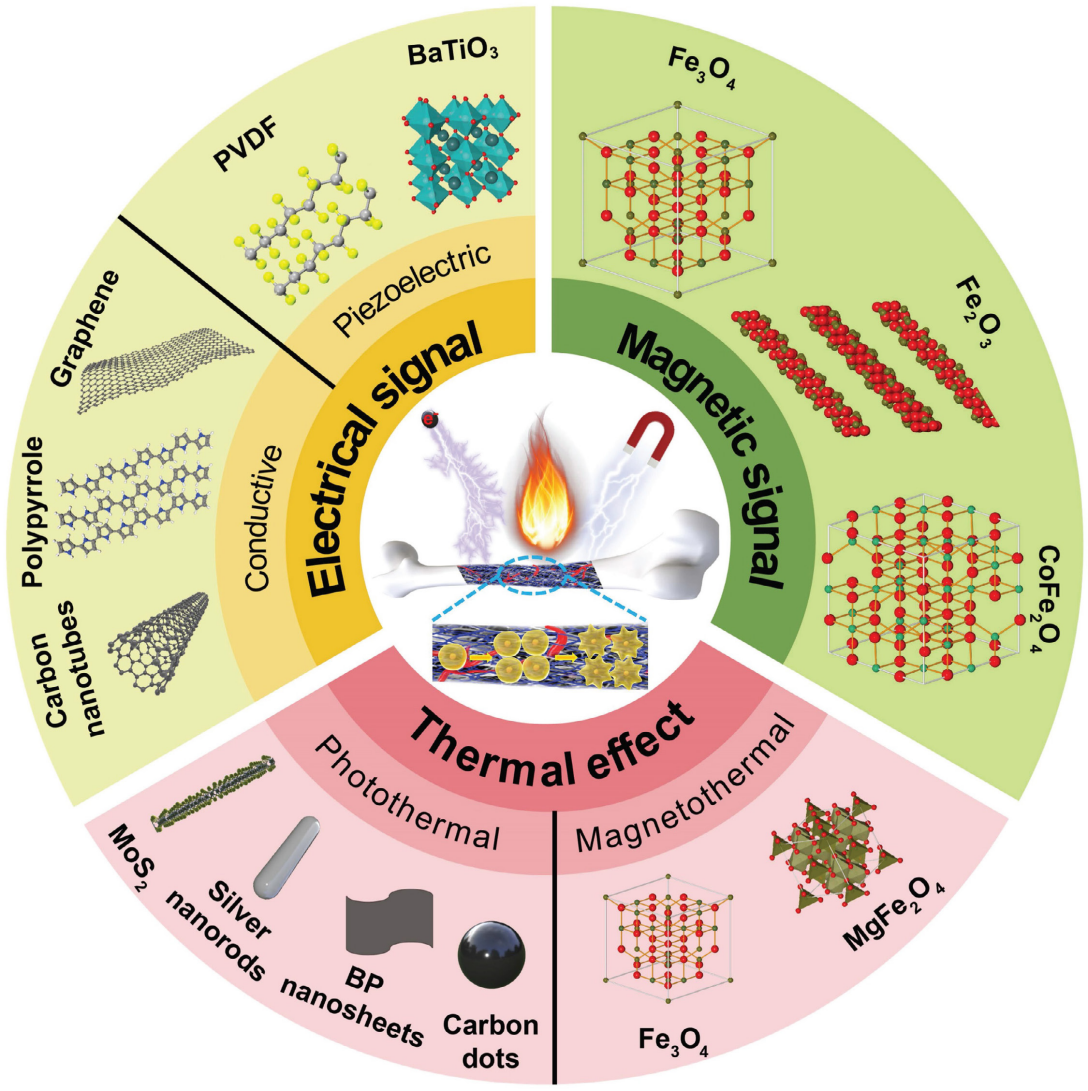
Schematic illustration of biomaterial-mediated physical cues (thermal effect, electrical and magnetic signals) for bone repair by remote actuation and on-demand activation.

Remote control of material properties through exogenous stimuli and on-demand activation of physical cues to guide the behavior of cells and organisms is important for developing bone tissue engineering. This review summarizes the generation of biomaterial-mediated physical cues (thermal effect, electrical and magnetic signals) and their impacts on bone repair processes, such as cell adhesion, migration, and differentiation (Figure 1). Material classification and corresponding outcomes of each physical cue are illustrated by *in vitro* and *in vivo* experimental results. This review will guide the development and clinical translation of intelligent stimulation-responsive materials for better bone regeneration outcomes.

## Biomaterials-mediated mild thermal effect

Mild thermal therapy is flourishing as a promising method for bone regeneration. Exogenous stimuli can activate bone repairing scaffolds on demand to generate a mild thermal effect, accelerating the healing of bone and surrounding tissues and achieving targeted therapy and precise regulation. NIR light and magnetic fields are ideal energy sources due to their tissue penetration, remote operability, and spatiotemporal accuracy. With the rapid development of nanotechnology, a series of biomaterials (photosensitizers and magnetic materials) that can respond to NIR light and magnetic fields to generate efficient thermal response and achieve favorable thermotherapeutic effects have emerged. After implantation of these biomaterials, exogenous laser irradiation (photothermal therapy, PTT) and magnetic field stimulation (magnetothermal therapy, MTT) can effectively promote osteocyte proliferation, tissue regeneration, and mineralization (Table 1). This section will discusses the impacts of the thermal effect on bone repair, followed by applications of photothermal and magnetothermal materials to treat bone defects.

### Impact of mild thermal effect

**Figure 2:**
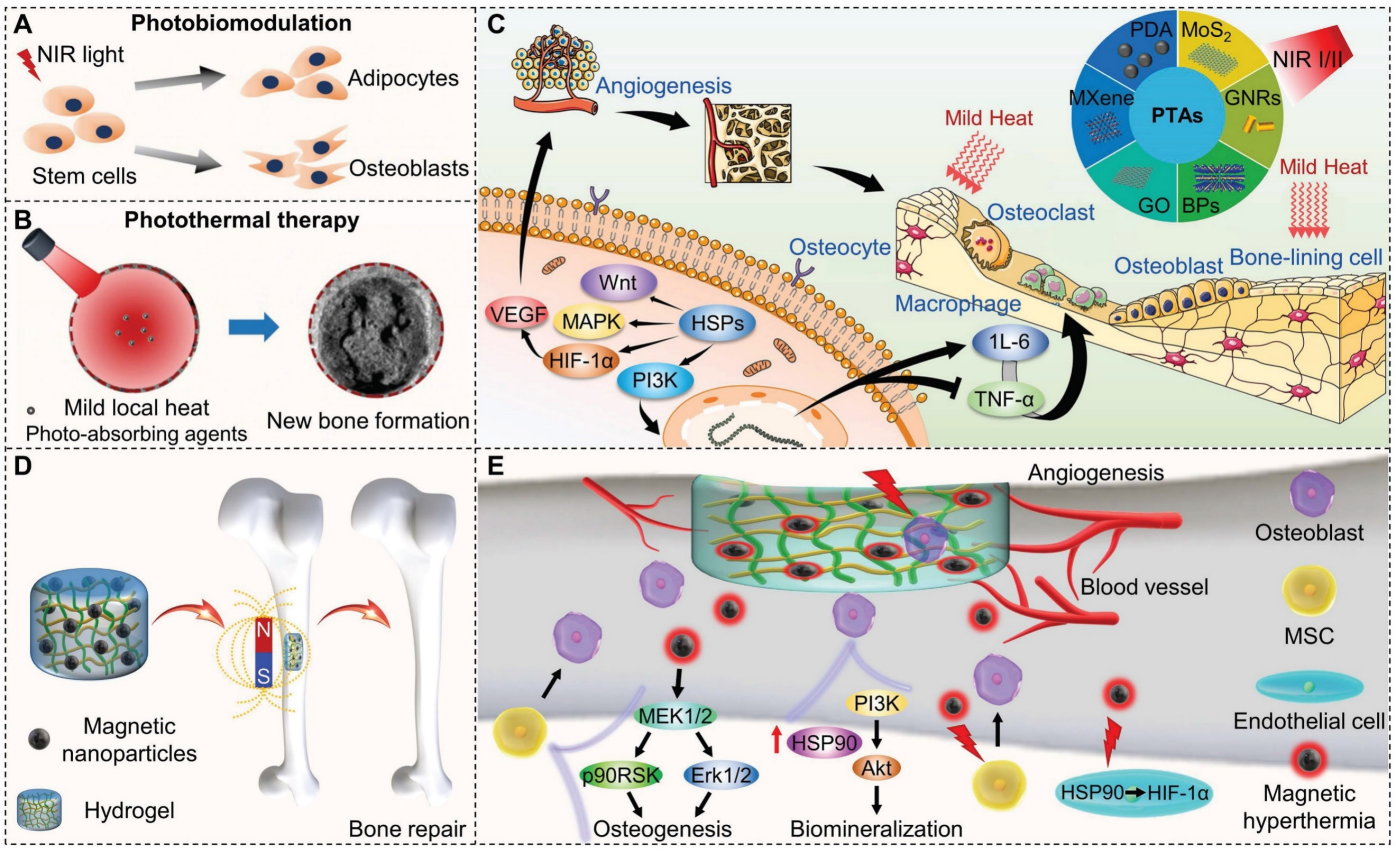
(A) Schematic of photobiomodulation under NIR light irradiation to regulate the attachment, proliferation, and differentiation of stem cells. Reproduced with permission from 42. Copyright 2020, Ivyspring International. (B) New bone formation by mild thermal effect of NIR light. Reproduced with permission from 42. Copyright 2020, Ivyspring International. (C) Possible mechanisms of mild thermal effect promoting bone regeneration and angiogenesis; commonly used photosensitizers for bone regeneration. Reproduced with permission from 14. Copyright 2023, Elsevier. (D) Schematic of magnetothermal effect promotes new bone formation. (E) Potential mechanisms of osteogenesis, biomineralization, and angiogenesis upon under magnetothermal effect.

Temperature control is important for hyperthermia-based therapies because it regulates physiological changes through elevated temperature. High temperatures (>45 °C) trigger DNA and protein denaturation or internal stress response, leading to apoptosis or necrosis, which are suitable for antibacterial and anti-tumor treatments. Mild thermal effect resulted by NIR light and magnetic fields can promote osteogenic differentiation and new bone formation. A low-intensity laser can stimulate the proliferation and osteogenesis of osteoblasts at the fracture site, promote cell migration and differentiation, reduce inflammatory response, and relieve pain through photobiomodulation therapy (Figure 2A) 42,43. Under NIR laser irradiation, the photosensitizers enriched in the bone defect site produce a mild thermal effect, which in turn promotes the differentiation of mesenchymal stem cells (MSCs), and the maturation and mineralization of osteoblasts (Figure 2B) 42. NIR light-mediated heat stress activates the expression of heat shock proteins (HSPs), subsequently influencing bone metabolic pathways and angiogenesis. This molecular response plays a crucial role in regulating the proliferation and differentiation of osteoblast-related cells (Figure 2C) 14. Mild hyperthermia induced by magnetic composites stimulated under a magnetic field with strong tissue penetration can effectively restore critical-sized bone defect (Figure 2D), because the thermal effect significantly promotes osteogenic differentiation and biomineralization of preosteoblasts via the HSP90-activated PI3K/Akt pathway (Figure 2E) 41.

In summary, the potential mechanisms of thermal effect on bone repair are listed as follows: (1) Warm stimulation helps to dilate blood vessels, improve local blood circulation, increase the supply of oxygen and necessary nutrients, and is beneficial to the healing of defects and surrounding soft tissues. (2) Heat stress induces the expression of osteogenesis-related proteins, such as alkaline phosphatase (ALP) and HSPs (especially HSP27, HSP47, HSP60, HSP70, and HSP90), which further promote immune remodeling, vascularization and osteogenesis. HSPs regulate the expression and conformation of osteogenic factors to adapt to a variety of physical and chemical stimuli such as hyperthermia, oxidative stress and ionizing radiation. When cells are exposed to high temperatures, the expression of HSPs is significantly increased, preventing protein denaturation and maintaining protein activity. Also, HSPs are involved in bone metabolism stages, such as bone resorption and bone regeneration, and play an important role in fracture healing 44. For example, HSP60 can promote the formation of osteoclasts via p38 MAPK and NF-κB pathways and affect the status and proliferation of osteoblasts 44. HSP27 is involved in specific regional and temporal expressions during tooth development 45. The upregulation of HSP90 by hyperthermia activates the downstream PI3K/Akt pathway and promotes bone regeneration 41. (3) Periodic mild photothermal stimulation can stimulate the immune system and related inflammatory processes, such as promoting macrophages to the anti-inflammatory M2 phenotype and increasing the expression of anti-inflammatory cytokines 46. (4) Thermal effect enhances angiogenesis through improving the expression of vascular endothelial growth factor and platelet-endothelial cell adhesion molecule (CD31) 47.

### Photothermal materials

The photothermal phenomenon refers to the absorption of photon energy by the photosensitizers accumulated in the lesion under laser irradiation. Then electrons are activated from the ground state singlet to the excited singlet state. Subsequently, the electron returns to the lower vibrational level after multiple collisions and releases heat during this vibrational relaxation 48,49. As a non-/less invasive treatment, PTT uses photosensitizers to convert light energy into heat for treatment and repair, demonstrating the characteristics of high spatiotemporal selectivity, slight side effects, and strong controllability 50,51. The hyperthermia generated during PTT is often used to ablate tumors and combat bacterial infections, while mild PTT shows potential for bone repair. The laser adopted here is mainly the NIR light located in the transparency window of biological tissues. Due to the limited depth of tissue penetration, NIR light-induced PTT is more suitable for the repair of superficial tissue damage. With the rapid development of nanotechnology, the laser has evolved from NIR-I (750-1000 nm) to NIR-II (1000-1350 nm) region for deeper tissue penetration, higher biosafety, and larger maximum allowable exposure 52-54. Photosensitizers with different absorption wavelengths have also been extensively studied. An ideal photosensitizer presents high photothermal conversion efficiency, good photothermal stability and biocompatibility. According to the type of materials, the commonly used photosensitizers in bone defect repair mainly include metals, metal oxides or sulfides, carbon materials, and other types of materials (such as black phosphorus (BP), polydopamine (PDA), etc).

#### Metallic photosensitizers

Metallic photosensitizers represented by gold nanomaterials show great potential in the biomedical field due to their easy synthesis, good biocompatibility, favorable stability, and outstanding photothermal effect. Notably, a study of the clinical pilot device using the local thermal effect of gold nanoparticles for prostate cancer treatment has demonstrated feasibility and safety in patients with localized low- or intermediate-risk prostate cancer, showing potential in clinical translation 55. In addition to eradicating tumors, the mild thermal effect of metallic materials can also promote bone tissue regeneration. Gold nanorods modified with endogenous proteins collected from autologous blood can improve biocompatibility and reduce immune inflammation and rejection. Under laser irradiation, the mild photothermal effect of gold nanorods promotes osteogenic differentiation of bone mesenchymal stem cells (BMSCs) through MAPK, Akt, Smad, and β-catenin pathways 26. Bismuth-doped glasses also show good photothermal performance. Under laser irradiation, these glasses improve biological activity and promote osteogenic cell proliferation, differentiation, and mineralization via photoinduced hyperthermia 56. Porous AuPd alloy nanoparticles, as thermotherapeutic agents, significantly accelerate cell proliferation and bone regeneration through PTT 27. Other metals (such as magnesium, iron, and manganese) and their alloys have attracted much attention in orthopedic implants due to their superior mechanical properties, biodegradability, and biological activity. Aside from stimulating the osteogenic differentiation of bone-forming cells by ionic products generated from metal degradation, the mild thermal effect caused by NIR light responsiveness also shows potential in bone regeneration 57,58.

#### Metal oxides or sulfides

Metal oxides or sulfides display improved osteogenic properties due to their mild photothermal stimulation. As a commonly used material in titanium implants, titanium dioxide (TiO_2_) is facile to prepare and has good biocompatibility. Although the absorption of TiO_2_ is mainly located in the ultraviolet band, its absorption and photothermal performance in the NIR region can be effectively improved by doping ions (such as Si and P) to form black TiO_2_ 59. The mild photothermal effect of black TiO_2_ and the release of doped ions triggered by NIR light can elevate the expression of osteogenic genes, regulate the behavior of osteoblasts, and also control inflammation, which can be used for peri-implantitis treatment 28,60,61. A photothermal double-layer biomimetic periosteum based on Nd_2_O_3_ presents excellent bone tissue regeneration function 29.

MoS_2_ nanosheets are a new class of layered two-dimensional metal sulfides. Due to the good biocompatibility and photothermal conversion efficiency, MoS_2_ nanosheets show promising prospects in bone tissue engineering. MoS_2_ and poly (ε-caprolactone) (PCL) nanofiber membranes obtained by electrospinning show favorable cell growth and osteogenesis properties, and the incorporation of MoS_2_ improves the mechanical properties of nanofiber membranes without being affected by degradation. Under the mild hyperthermia effect induced by NIR light, these nanofiber membranes promoted the growth of BMSCs *in vitro* and helped to repair the rat tibial bone defect 30. In another report, BMSCs were seeded into photothermal MoS_2_-biotin-sucralose gelatin scaffolds to improve osteogenic activity. Subsequently, an osteoinductive extracellular matrix was used to cover these scaffolds to simulate the biomimetic microenvironment. The obtained scaffolds significantly induced bone regeneration in rats with bone defects 31.

Notably, the photothermal properties of some metal composites (such as SrCuSi_4_O_10_ 62, CuS 32, and WS_2_ 63) can be extended to the more prominent NIR-II region, which is beneficial for repairing deep tissue defects. CuS nanoparticle-PEG soft hydrogel-coated 3D hard PCL scaffolds demonstrate excellent photothermal properties. Under 1064 nm laser irradiation, the mild thermal effect of 42 ± 0.5 °C effectively promoted the osteogenic differentiation of BMSCs and improved bone regeneration of rat tibial defect 32. Cu^2+^ and PO_4_^3-^ have been reported to have excellent tissue regeneration and osteogenesis abilities, and CuP-based bone-targeting nanosystems also exhibit NIR-II photo-responsiveness. Under 1064 nm laser irradiation, the resulting thermal effect effectively inhibits bone resorption caused by massive osteoclast differentiation, providing a promising treatment for osteolytic bone metastasis 64. The CD/WS_2_ heterojunctions, formed by the electrostatic assembly of CDs with WS_2_, present enhanced NIR-II absorption and photothermal conversion efficiency and exhibit good photothermal effects under 1064 nm (0.6 W cm^-2^) laser irradiation after penetrating 10 mm-thick tissue. Periodic low-intensity laser irradiation and warm environment stimulate the expression of bone-related genes such as HSPs and accelerate osteoblast differentiation 63.

#### Carbon materials

Carbon materials (like MXene, carbon dots (CDs), graphene oxide (GO), carbon nanotubes (CNTs), etc.) are promising in the field of bone repair owing to their excellent biocompatibility and photothermal properties. The MXene-based material can attenuate local immune responses while promoting new bone formation by mild thermal stimulation 33,65,66. CDs doped in drug-loaded bone repairing materials can synergistically stimulate angiogenesis, promote osteoblast differentiation, and accelerate bone repair through NIR laser-triggered drug release and mild thermal effect 67. CD-based composites can also generate photothermal effects in the NIR-II biowindow for osteosarcoma therapy and bone tissue regeneration 63. Yang *et al*. developed a temperature-controlled multifunctional nano-hydroxyapatite (HA)/GO/chitosan (CS) scaffold for tissue repair after osteosarcoma resection. Under 808 nm laser irradiation, the hyperthermia generated by the scaffold (48 ℃) could effectively kill osteosarcoma cells while promoting osteogenesis of human BMSCs through a mild thermal effect (42 ± 0.5 °C) 34. Another study reported that the mild thermal effect triggered by CNT and NIR laser irradiation (42 ℃, 15 min per day) significantly up-regulated the expression of osteoinductive genes (ALP, osterix, and osteocalcin), thereby promoting mineral deposition and bone repair 35. Thermosensitive hydrogels combined with carbon particles were injected into irregular bone defects and underwent sol-gel transition by body temperature, which could be used for conformal therapy. Results of the 8-week treatment of skull bone defect in rats with these hydrogels showed that the bone volume/total volume ratio reached 76.2%, significantly higher than 23.9% in the control group, demonstrating the effectiveness of this strategy 68.

#### Other photothermal materials

PDA, BP, and indocyanine green are widely used photothermal agents in bone repair, among which PDA could also reduce inflammation caused by reactive oxygen species (ROS) 69. BP is a two-dimensional elemental material possessing a black metallic luster, a unique folded layered structure, and extraordinary optical and electronic properties desired by biomedical application 70,71. BP exhibits good biocompatibility, high photothermal conversion performance, and suitable degradation ability. Under NIR laser irradiation, BP can induce a mild photothermal effect and promote bone regeneration by activating HSP-mediated signaling pathways 72. Furthermore, BP is prone to decompose into phosphate ions in the presence of oxygen and/or water to extract Ca^2+^ from the physiological environment, thus promoting mineralization 38,73. It has been reported that poly(lactic-*co*-glycolic acid) (PLGA) containing only 0.2 wt% BP (BPs@PLGA) shows an efficient NIR photothermal response, even when covered with 7 mm thick biological tissue 39. The mild thermal effect and the release of elements required for bone formation endow the BP-based nanocomposite scaffolds with good osteogenic activity. Moreover, introducing other functional units, such as antibacterial agents and exosomes, can further enrich the functions of this system.

**Figure 3:**
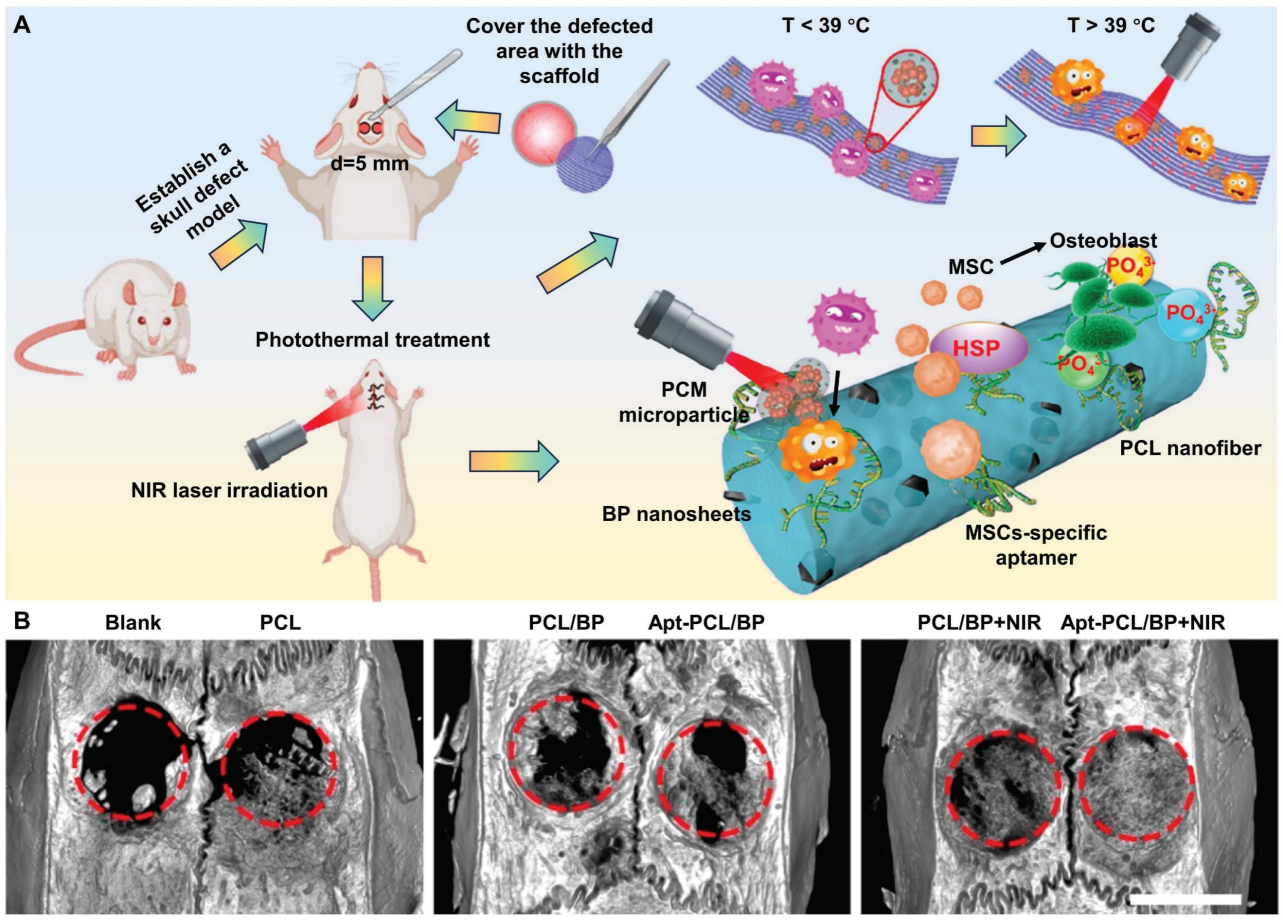
(A) Schematic illustration of a multifunctional scaffold for bone regeneration that kills bacteria, recruits endogenous MSCs, and maintains a mild photothermal effect. (B) Micro-CT 3D reconstruction of the repaired bone tissues at 8 weeks after implanted with different types of scaffolds in rat calvaria defect model. Scale bar = 5 mm. Reproduced with permission from 40. Copyright 2023, American Chemical Society.

As shown in Figure 3A, to selectively recruit MSCs to the injured site, the surface of electrospun PCL nanofibers doped with BP nanosheets is modified with the nucleic acid aptamer Apt19S, which can specifically recognize MSCs. Subsequently, the microparticles of phase change materials (PCMs) loaded with antibacterial drugs are deposited on the surface of these scaffolds. Under laser irradiation, the photothermal effect of BP activates the solid-liquid phase transition of these scaffolds (T > 39 °C), triggering drug release, thereby killing bacteria and preventing infection. The mild thermal effect-mediated upregulation of HSPs and biodegradation of BP synergistically promote osteogenic differentiation and biomineralization of MSCs. Micro-CT 3D reconstruction of bone tissue showed that new tissue formed at the edge and center of the defect in the Apt-PCL/BP group combined with NIR irradiation after 8 weeks of scaffold implantation, verifying the efficacy for bone regeneration *in vivo* (Figure 3B). This strategy provides valuable information for the rational design of biomimetic scaffolds 40.

### Magnetothermal materials

The magnetothermal effect refers to the local thermal effect caused by the resonance between the spin magnetic moment of the magnetic material gathered in the lesion and the external AMF. MTT has advantages such as high flexibility, non-/minimal invasiveness, and easy remote operation 74,75. The depth and intensity of treatment can be controlled by adjusting the strength and frequency of AMF. Therefore, MTT is more beneficial for treating bone defect-related diseases because the tissue penetration depth of the magnetic field is deeper than that of NIR light 76.

The properties of exogenous AMF and MNPs to promote MSC proliferation and osteogenic differentiation through targeted thermal effect have attracted much attention 77-79. For instance, a US Food and Drug Administration (FDA)-approved polymethyl methacrylate (PMMA) material contains Fe_3_O_4_ nanoparticles to produce liquid phase PMMA-Fe_3_O_4_ bone cement, which is then injected into bone tumors to fill bone defects. The cured cement provides mechanical support for bone defects and alleviates pain, and the warm effect generated by the external AMF can repair bone defects 80. Low melting point metals exhibit a strong magnetothermal effect upon AMF stimulation due to their high electrical and thermal conductivity. Bismuth alloy has the advantages of low melting point, good biocompatibility, strong bone affinity, and stability in the bone defect site for up to 210 days. Combined with the magnetothermal effect generated by AMF stimulation, the alloy can be used for long-term bone defect repair and analgesia 81. Furthermore, the MNPs heat the scaffold and significantly accelerate its degradation with AMF, suggesting the benefits of the development of magnetically controlled degradation implants 82.

Superparamagnetic nano Mn-Zn-Cu-Gd ferrites with low Curie temperature (65 ℃) were incorporated into bone cement matrix to avoid heat damage to normal tissues, achieve self-controlled hyperthermia, and promote the mineralization of osteoblasts 83. The optimized osteoinductive nanoparticles-hydrogel composite prepared by embedding the Arg-Gly-Asp (RGD)-coated magnetic iron oxide nanoparticles (CoFe_2_O_4_@MnFe_2_O_4_) in agarose had a significant magnetothermal effect. The combination of the composite with the highly tissue-penetrating AMF produced a temperature of 41-42 ℃, significantly promoting the osteogenic differentiation and biomineralization of preosteoblasts via the PI3K/Akt pathway activated by HSP90 41. However, besides the adverse effects of magnetic fields on the body, the potential thermal damage to normal tissues and the long-term toxicity of materials are also important challenges in the clinical translation of MTT.

## Biomaterials-mediated electrical signals

**Figure 4:**
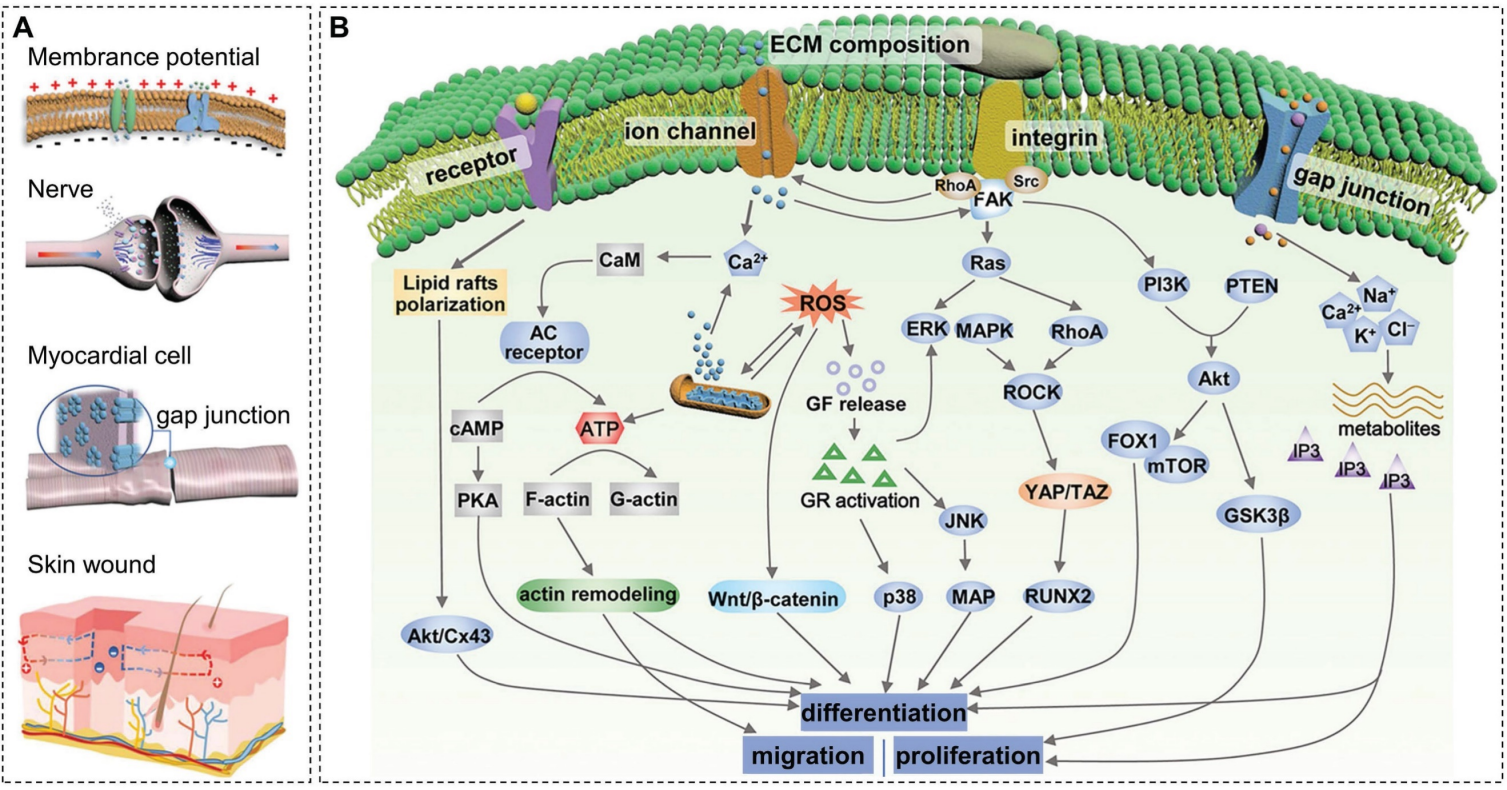
Schematic of (A) endogenous bioelectricity and (B) possible pathways involved in the biological response to electrostimulation. Reproduced with permission from 84. Copyright 2021, Wiley-VCH.

Bioelectricity is a key physiological activity of living organisms. Endogenous electric fields exist in several organs, such as skin, heart, nerve, and bone (Figure 4A), essential for maintaining normal physiological activity 84. When bone tissue is damaged, the potential of the defect site decreases. Restoration of the local bioelectrical microenvironment by transmitting electrical signals through the damaged tissue takes time, and it is difficult for osteoblasts to migrate from the edge of the defect area to the center 85. Under such circumstances, electroactive biomaterials serve as scaffolds for cell adhesion and structural support and also help to reconstruct the electrophysiological microenvironment of bone tissue, enhance local electrical stimulation, and regulate cell/tissue behavior and function. The ideal electroactive material must mimic the layered structure of natural bone, generate and/or transmit electrical signals, and regulate stem cell fate while being fully compatible and degradable in the physiological environment. Current research on electroactive materials mainly focuses on conductive materials, piezoelectric materials, and other electrically responsive biomaterials (such as magnetoelectric and photoelectric materials). Among them, conductive and piezoelectric materials are widely used in bone repair. Some representative data for conductive and piezoelectric materials are summarized clearly in Table 2 to facilitate comparison.

### Impact of electrical signals

Bone is considered a natural piezoelectric composite material, which generates piezoelectric phenomena and physiological potentials due to the electronic displacement of local electric fields caused by the mechanical deformation of collagen (COL) structures 100,101. The piezoelectric effect in natural bone was first observed by Japanese scientists Fukada and Yasuda in 1957 102 and later confirmed at the molecular level by infrared spectroscopy by Lipieca et al. in 2012 103. It has been reported that the piezoelectric constant (d_33_) of bone tissues is about 0.7-2.3 pC N^-1^ and the physiological potential ranges from -60 to -100 mV 104. According to Wolf's Law, bone can constantly adjust its shape, strength, and density to resist external forces, and a persistent lack of force will cause bone resorption, resulting in decreased bone density 105. With the change of mechanical stress, the potential gradient along COL fibers provides local stimulation to bone regeneration cells such as osteoblasts and osteocytes, which help to maintain the normal metabolism and physiological activities of bone tissues.

As an inducing factor, electrical stimulation plays an important role in bone maturation, remodeling, and reconstruction. The piezoelectric potential of bone generates negative charges under physiological loading, further stimulating the proliferation and differentiation of osteoblasts and thereby promoting bone regeneration 106. The higher pressure load generates a higher piezoelectric potential in a specific range. Figure 4B displays the underlying mechanisms of electrical stimulation of osteogenesis, which may include activation of cell membrane ion channels, focal adhesion-associated mechanotransduction signaling axis, increase of local blood flow, and regulation of cell signal transduction pathways 16,84. Specifically, ion channels are hydrophilic microchannels that allow ions to pass through the cell membrane selectively. Upon stimulation of electrical signals generated by mechanical stress, Ca^2+^ channels on the surface of the cell membrane open, and Ca^2+^ influx leads to increased intracellular Ca^2+^ concentration, which, in turn, activates calcineurin and calmodulin. Calcineurin reacts with the phosphorylated nuclear factor of activated cells to generate the dephosphorylated nuclear factor NF-AT, which then translocates into the nucleus. NF-AT cooperates with other transcription factors to regulate gene transcription, promotes the synthesis of transforming growth factor-β (TGF-β) and bone morphogenetic protein (BMP), and regulates the production of extracellular matrix and cell metabolism 107. In addition, Ca^2+^ can activate the blocking activity of gelsolin, release actin, and promote cell migration 108.

On the other hand, electroactive biomaterials enhance integrin receptor binding and aggregation by altering the conformation of adsorbed fibronectin through surface charges, accelerating the formation of focal adhesion complexes. Focal adhesion kinase (FAK) is activated by focal adhesion aggregation, which triggers the mechanotransduction signaling axis and promotes osteogenic differentiation through YAP/TAZ transcriptional coactivation of key genes involved in osteogenesis 109,110. Electrical stimulation can promote vasodilation, increase the permeability of the vessel wall, and deliver white blood cells and oxygen to the wound to accelerate bone healing. Also, electrical stimulation promotes bone regeneration by activating signaling pathway-mediated osteogenic effects, such as enhanced expression and release of growth factors.

### Conductive materials

Carbon-based nanomaterials, metallic materials, and conductive polymers are widely used conductive materials for bone repair. Conductive materials amplify and deliver endogenous electrical signals to cells/tissues upon external electrical stimulation. This electrical signal can guide the osteogenic differentiation of bone-related cells and also change the surface charge density of the conductive matrix to promote mineral nucleation and growth, thus accelerating cell proliferation and differentiation.

#### Carbon-based nanomaterials

Carbon materials such as CNTs and graphene are ideal conductive biomaterials studied in bone regeneration, and CDs, nanodiamonds, and fullerenes have shown great potential 111. Although these nanomaterials are mainly composed of the same carbon element, the differences in structure, surface functionality, and size endow them with different characteristics. Besides the good mechanical properties, biological activity, biocompatibility, and chemical stability, carbon-based nanomaterials also demonstrate a large specific surface area and high electrical conductivity, which can be used to improve the mechanical properties and electrical conductivity of bone repairing material and stimulate bone regeneration. Incorporating CNTs into the polymer has been reported to effectively improve the tensile and compressive modulus of the composite scaffolds 112.

The similar surface roughness of CNTs to native COL enables them to positively affect cell adhesion, proliferation, and differentiation 113,114. CNTs have a small diameter, a high specific surface area, and an ordered arrangement of internal carbon atoms through strong sp2 bonds. Electrons move freely in the tube to form one-dimensional conductive channels with a conductivity as high as 10^4^ S cm^-2^, which is ten thousand times that of copper 115. Under direct current stimulation of 100 μA, biodegradable poly-DL-lactide (PLA) nanofibers embedded with multi-walled CNTs fabricated by electrospinning can guide cellular elongation and proliferation, showing great potential in bone regeneration and fracture healing 116. Exogenous electrical stimulation combined with conductive scaffolds can promote osteoclast formation and the expression of nuclear factor κβ ligand RANKL, thus significantly promoting angiogenesis and mineralization and dominating the process of bone remodeling 86. Furthermore, CNT-containing scaffolds exhibit sensitive electrochemical response signals to osteogenic differentiation and tissue mineralization at the cellular and animal levels, which can be used for long-term noninvasive and continuous monitoring of osteogenic differentiation levels 117.

Graphene is one of the strongest materials known. The formation of conjugated large π bonds by carbon atoms in the structure endow it with high electrical conductivity. Homogeneous doping of PDA-reduced GO in the network enhances the mechanical properties of the network while providing a conductive pathway that allows it to act as an electroactive matrix to transmit electrical signals and regulate the proliferation and morphology of C2C12 cells 118. BP is a graphene-like material with high electrical conductivity and topology due to its anisotropy, which is also an ideal conductive material for bone regeneration 119,120. In particular, the large specific surface area of carbon-based biomaterials can be loaded with a variety of bioactive substances (growth factors, drugs, etc.) to synergize with the electrical activity to promote osteogenesis. Nonetheless, carbon materials are difficult to disperse uniformly, and agglomeration may reduce mechanical properties. The long-term safety of nondegradable carbon nanomaterials *in vivo* also requires further investigation.

#### Metallic materials

Gold and silver are commonly used conductive biomaterials in bone regeneration. Gold nanoparticle-doped hybrid hydrogel scaffolds could increase connexin 43 expression in neonatal rat cardiomyocytes, indicating that electroelastic scaffolds can potentially improve cardiomyocyte function 121. Furthermore, silver nanoparticles could work as a conductive phase to enhance the electrical activity of other materials to promote bone repair 122. The moderate dissociation in the physiological microenvironment is advantageous for metallic bone repairing materials because some metal ions are structural components of osteogenic enzymes and proteins. Introducing Fe^3+^ into hydrogels could significantly improve its conductivity as strain sensors in tissue repair 123. However, the uncontrolled release of metal ions caused by wear or corrosion *in vivo* is prone to trigger inflammatory cascades. In constrast, metallic glass has higher wear and corrosion resistance. Upon electrical stimulation, the electrical signal amplified through gelatin methacryloyl gel, containing conductive palladium-based metallic glass submicron wires, significantly promotes the formation, contractility, and metabolic activity of mouse myoblast C2C12 myotubes 89.

#### Conductive polymers

Conductive polymers, mainly polypyrrole (PPy), polyaniline (PANI), polythiophene, and poly(acetylene), are a new class of intelligent materials with long-range π electronic backbone structures formed by alternating arrangement of localized carbon-carbon single bonds and less localized double bonds. Under the stimulation of an external electric field, the internal charge carriers move along the conjugated π bonds, thus achieving the directional transfer of electrons, manifested as electrical conductivity. In the biomedical field, conductive polymers have attracted much attention due to their strong structural flexibility and redox reversibility compared with conductive carbon nanomaterials and metallic materials. Furthermore, according to the type and amount of dopant used, the conductivity of PPy and PANI can usually reach 10-10^3^ S cm^-1^ and 30-200 S cm^-1^, which is close to that in human tissues 124. There are various methods for preparing and modifying conductive PANI, and selecting appropriate stabilizers helps improve integrative performance. The PANI-based films prepared with sodium dodecyl sulfate as a stabilizer were reported to have high electrical conductivity and good cytocompatibility, as well as non-irritant to the skin 125.

**Figure 5:**
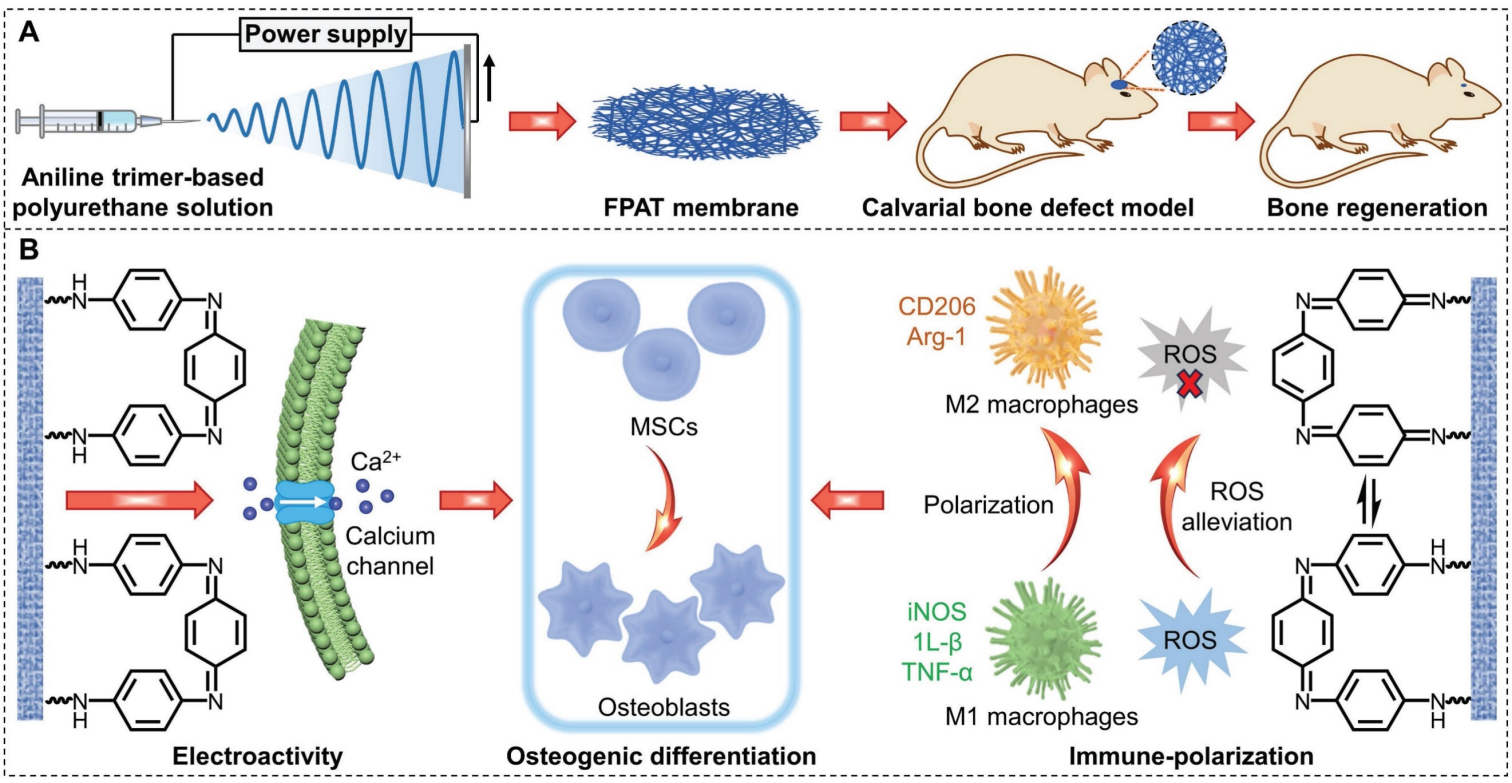
(A) Schematic of FPAT membranes prepared by electrospinning for calvarial defect repair. (B) The electroactive FPAT membranes restore the electrophysiological microenvironment, regulate site-specific cellular behaviors and promote osteogenic differention.

As shown in Figure 5A, fibrous aniline trimer (AT)-based polyurethane (FPAT) membranes prepared by electrospinning were used for calvarial defect repair. The FPAT electrospinning membranes with good electrical activity could increase Ca^2+^ concentration in MSCs, up-regulate the Ca^2+^ signaling pathway, promote osteogenic differentiation, and accelerate bone regeneration. In addition, the redox property of FPAT membranes allow them to remove ROS from the organism, which, in turn, promotes bone repair by inducing macrophage polarization toward the M2 phenotype (Figure 5B). Fluorescence staining indicates that electroactive membranes can stimulate the accumulation of intracellular Ca^2+^ and remove excessive intracellular ROS. The bone regeneration areas after FPAT membrane treatment are significantly larger than in other groups, indicating the superior bone regeneration ability with FPAT membranes *in vivo* 90. In order to grasp the progress of bone repair, the combination of conductive materials with flexible electronic devices may be a potential approach 126. However, there are few *in vivo* applications of conductive polymers, which may be due to their poor degradation, easy loss of conductivity, and physiological toxicity caused by small molecule residues. Dependence on external power supply, complex operation, and low efficiency are also major obstacles to practical applications of conductive materials.

### Piezoelectric materials

The piezoelectric effect refers to the physical phenomenon of generating potential difference between the two ends of a dielectric material under a mechanical load or deformation. The surface charge arises from the rearrangement of dipoles triggered by an external force, and the material can maintain a charged state for a period after polarization without continuous stimulation. Therefore, compared with conductive materials, piezoelectric materials are more suitable for clinical use. Due to the outstanding performance of bioelectric signals in repairing damaged tissues and restoring cellular functions, piezoelectric materials have attracted much attention in orthopedic applications 127,128. According to the material type, the commonly used piezoelectric materials in bone repair mainly include ceramics and polymers, among which piezoelectric polymers include natural and synthetic types.

#### Piezoelectric ceramics

Barium titanate (BaTiO_3_) 129, lithium niobate 130, and potassium sodium niobate 91 are well-known biological piezoelectric ceramics. The BaTiO_3_ with tetragonal perovskite structure presents a high piezoelectric constant (d_33_ > 190 pC N^-1^), which generates polarization under an external force or a strong direct current electric field, separating the internal electrons from the holes and generating a voltage 131. Although BaTiO_3_ is not degradable *in vivo*, it still shows good biocompatibility and osteogenic ability and is an excellent piezoelectric material for hard tissue repair. BaTiO_3_ is usually combined with nondegradable polymers (such as polyvinylidene fluoride (PVDF), poly(vinylidene fluoridetrifluoroethylene) (P(VDF-TrFE)), and polyamide 12) 94,132,133, degradable polymers (PLA, for instance) 95 and other inorganic materials (HA and Ti6Al4V, etc.) 104,134 because of its poor processing property, mechanical strength, and toughness.

The piezoelectric effect generated by polarized BaTiO_3_/Ti6Al4V scaffold has been demonstrated to promote macrophage M2 polarization and immunomodulatory osteogenesis in MC-3T3 osteoblasts 134. The piezoelectric BTCP ceramics prepared by two-step sintering of BaTiO_3_ and β-tricalcium phosphate (β-TCP) form different types of charges through high-voltage polarization, thereby synergistically regulating cellular immunity and osteogenic function to guide bone healing. The negatively charged, polarized BTCP (BTCP-) promotes protein adsorption and extracellular Ca^2+^ influx of BMSCs, increases integrin α5β1, P-FAK, and P-ERK protein expression, and enhances osteogenic differentiation through FAK/ERK and BMP/Smad signaling pathways. The polarized BTCP ceramics with a positive surface charge (BTCP+) significantly inhibit the polarization of pro-inflammatory (M1) macrophages, alleviate the local inflammatory response, and form an immune microenvironment conducive to osteogenesis 135.

It has been reported that polarized BaTiO_3_ enhanced calcium phosphate deposition 136, and the introduction of the piezoelectric phase promoted the adhesion and proliferation of mouse fibroblast L929 and human osteoblast SaOS2 cells 137. BaTiO_3_ possesses a superior intrinsic ability to maintain charged surfaces 95,138. The original piezoelectric coefficient (d_33_) of the nanocomposite membranes, containing BaTiO_3_ nanoparticles embedded within a P(VDF-TrFE) matrix and immersed in the culture medium for 4 weeks, was maintained more than 90% 129. After corona polarization, the surface potential of the flexible nanocomposite membrane prepared by BaTiO_3_ nanoparticles, PDA, and PVDF reached -76.8 mV, consistent with the endogenous biological potential level. Moreover, it could maintain over half of the original surface potential at the bone defect site for 12 weeks 94.

When BaTiO_3_ was combined with conductive silver and shape memory polymer of acrylate epoxidized soybean oil, the scaffolds demonstrated high accuracy, customizability, shape memory, and piezoelectric properties, which could effectively promote bone regeneration 139. HA, one of the main components of bone tissue, has also been shown to have piezoelectric activity due to its non-centrosymmetric monoclinic crystal structure 140,141. However, the polarization conditions of HA-based ceramics are relatively harsh, usually requiring high direct current intensity (approximately 1-2 kV cm^-1^) and high temperature (about 300-500 ℃), which is an important reason to limit the development of piezoelectric HA to practical applications 142,143.

#### Piezoelectric polymers

Biopiezoelectric polymer-based composites have promising applications in regenerative medicine and tissue repair. Currently, the commonly used piezoelectric polymers for bone repair include synthetic (PVDF, PLLA, poly[(*R*)3-hydroxybutyrate] (PHB), etc.) and natural polymers (like COL, cellulose, and chitin). PVDF is a semi-crystalline polymer composed of five known crystalline phases, α, β, γ, δ, and ε, and is also one of the most widely studied piezoelectric polymers 144. Among the crystalline structures, the polar β phase of PVDF shows the strongest piezoelectric activity due to the high density of dipoles in the same direction. β-phase PVDF has excellent biocompatibility, piezoelectric properties, and a large specific surface area, which can fit the COL fiber structure of the natural extracellular matrix and has been demonstrated to stimulate osteogenic differentiation. The better piezoelectric performance of PVDF with a higher content of β-phase has been shown 145.

Currently, there are two commonly used strategies to improve the β-phase content of PVDF. On one hand, introducing nanoparticles into the system could induce β phase nucleation 146. The uniform distribution of the PDA-modified BaTiO_3_ nanoparticles in the PVDF matrix increased the β-phase content from 46% to 59%, raising output voltage by 356% 132. The β-phase content could also be enhanced by adding TiO_2_ powder to the PVDF electrospinning solution with a stretching effect on the PVDF chain 145. On the other hand, the polarization and stretching effect of the high voltage electric field during electrospinning could promote the transformation of the non-polar α phase to the polar β phase of PVDF, and the content and crystallinity of the β phase could be effectively improved by adjusting the processing parameters 96.

PVDF is usually combined with other bioactive substances, such as hydrogel, nanocrystals, and GO to enhance piezoelectric effect and achieve excellent electrical signal stability and biological regulation. Piezoelectric PVDF composites could significantly promote cell adhesion, proliferation, and differentiation through surface electrical signals and enhance angiogenesis and osteogenic activity 146-149. By modifying ZIF-8 nanocrystals in hierarchically architected PVDF foam, the resulting PVDF/ZIF-8 piezoelectric foams can realize a stable voltage output of up to 10 V without polarization. Piezoelectric PVDF affects the molecular trajectory of Zn^2+^ released from ZIF-8, up-regulates oxidative phosphorylation and ATP-coupled cation transmembrane transportation in vascular endothelial cells, thus enhancing the uptake of Zn^2+^ by cells, and guiding vascularized bone regeneration through micro-current stimulation and Zn^2+^ enrichment. PVDF and PVDF/ZIF-8 foam particles were mixed with commercially available bone grafts in equal proportions and filled in the box-shaped defect site of the mouse femur. The presence of PVDF foam-based particles significantly improved the electrical properties of the femur. After 4 weeks, the bone healing outcome of PVDF/ZIF-8-M combined with the piezoelectric condition was obvious compared to that of the non-piezoelectric PVDF/ZIF-8-M group, which was slightly worse. According to micro-CT results, PVDF/ZIF-8-M foam significantly promoted bone repair and avoided fractures under piezoelectric conditions 148. Besides, piezoelectric PVDF-based copolymer (P(VDF-TrFE)) could recruit stem cells and promote osteogenic differentiation by mimicking periosteal structure/function and physiological loading conditions 97,98.

However, it should be noted that BaTiO_3_ and PVDF-based polymers are difficult to degrade *in vivo* and usually require secondary surgical removal after tissue repair is completed. Therefore, it is necessary to use biodegradable piezoelectric biomaterials. PLLA, PHB, and poly[3-hydroxybutyrate-*co*-3-hydroxyvalerate] (PHBV) polymers meet all requirements. PLLA is a semi-crystalline polymer containing a C=O dipole, and its thermodynamically stable conformation is an α-crystalline phase with helical polymer chains, while the piezoelectric properties stem from the β-type structure formed by C=O rearrangement after polarization 150. As a widely used biodegradable polymer, PLLA scaffold can generate a weak but stable electric field to promote cell migration/recruitment under external force stimulation. The experimental results of rabbit knee joint injury treated with PLLA nanofiber scaffold showed that piezoelectric effect could effectively stimulate cartilage regeneration 99. However, the piezoelectric properties of PLLA are weak and their relation with bone growth promotion remains unclear and requires further investigation.

PHB and PHBV are aliphatic polyesters widely distributed in the cytoplasm of prokaryotic cells, especially bacteria, which can completely degrade into non-toxic products *in vivo* 151. The existence of asymmetric carbon atoms associated with the polar C=O group in the structure endows PHB and PHBV with piezoelectric properties comparable to those of biological bone. Therefore, PHB- and PHBV-based piezoelectric scaffolds can fill bone tissue defects and stimulate new bone formation under dynamic mechanical conditions 152. Nevertheless, the insufficient mechanical strength and uncontrollable degradation rate of PHB and PHBV limit their use.

Natural polymers such as polysaccharides (CS, sodium alginate, and hyaluronic acid, for instance) and proteins (COL, gelatin, fibrin, etc.) are also widely used in bone repair due to their good material accessibility and biocompatibility. Especially, natural polymeric materials with hierarchical structures such as COL, cellulose, and chitin possess piezoelectric properties with a piezoelectric constant d_14_ of approximately 0.2-2 pC N^-1^, 0.1-0.2 pC N^-1^, and 0.2-1.5 pC N^-1^, respectively 153. Natural polymers have superior biological affinity due to their properties similar to the extracellular matrix. The piezoelectric properties can be amplified by molecular engineering for applications in various fields such as bone regeneration. In 2009, Jolandan and Yu isolated a single COL-I fiber from a bovine Achilles tendon and found that these fibers mainly behave as shear piezoelectric materials with a piezoelectric constant d_15_ of about 1 pC N^-1^ 101. The highly oriented and patterned structure of COL-I in bone and its ability to respond to mechanical loading confer piezoelectricity on bone tissue. Under the action of shear force, the COL fibers slide against each other, generating a piezoelectric charge, which in turn stimulates osteoblasts, induces mineralization and promotes bone formation 154. The deposition of HA is an important step in bone repair. It has been reported that the piezoelectric dipole generated by deformed COL can promote HA deposition through electrochemical methods without catalyst 100.

### Other electroactive materials

In recent years, the application of other electroactive materials such as magnetoelectric and optoelectronic materials in bone regeneration has attracted much attention. Magnetoelectric materials are composed of piezoelectric materials and magnetostrictive components. Electrical properties of these materials can be adjusted by external magnetic fields. Magnetostrictive materials move and rotate between magnetic domains in response to an external magnetic field, thus stretching and deforming the material in length and volume, which in turn stimulates piezoelectric materials to generate electrical signals 155. Liu *et al*. fabricated polarized flexible CoFe_2_O_4_@BaTiO_3_/P(VDF-TrFE) core-shell particle-incorporated composite membranes with high magnetoelectric conversion efficiency. Under the stimulation of magnetic field, the magnetic driving force on the CoFe_2_O_4_ magnetic core transferred to the BaTiO_3_ shell and increased its charge density, and enhanced the β phase transition of P(VDF-TrFE) matrix, leading to increased surface potential and osteogenic activation. Cranial defect experiments in male rats showed that repeated magnetic field applied to the composite membranes enhanced bone defect repair even under coexisting inflammatory conditions 156.

Photoelectric materials can absorb light energy and undergo photoelectron conversion reactions to produce electric energy. Three-dimensional biomimetic scaffold integrated with thin-film silicon-microstructures are able to regulate the membrane potential and intracellular calcium dynamics of stem cells through infrared light-induced electrical signals, and effectively promote cell proliferation, differentiation, and osteogenesis 157. Nanogenerator has the characteristics of converting mechanical energy into electrical energy, but it is mainly used in energy harvesting, energy storage, health monitoring and other fields, and its application in bone repair needs to be further developed 158,159.

### Biomaterials-mediated magnetic signals

As a non-/less invasive, safe, and convenient physical stimulation, the magnetic signal has significantly enhanced wound healing, bone defect repair, and osteoarthritis relief. Magnetic signals promote osseointegration and bone regeneration by activating cell surface receptors, stimulating the expression of bone-related genes, promoting cell adhesion, proliferation, migration, and differentiation, and inhibiting osteoclast formation 160. However, magnetic stimulation of cells by remote modulation of external magnetic field alone is insufficient. The combination of magnetic field and magnetic implants plays a special multifunctional synergistic role in clinical bone repair. The following section reviews the recent progress of magnetic signals mediated by biomaterials in bone tissue engineering. First, we review the impacts of magnetic signal on osteogenesis, and then we introduce the cutting-edge research on magnetic strategies based on MNPs and scaffolds to improve cell and tissue repair functions (Table 3).

### Impact of magnetic signals

**Figure 6:**
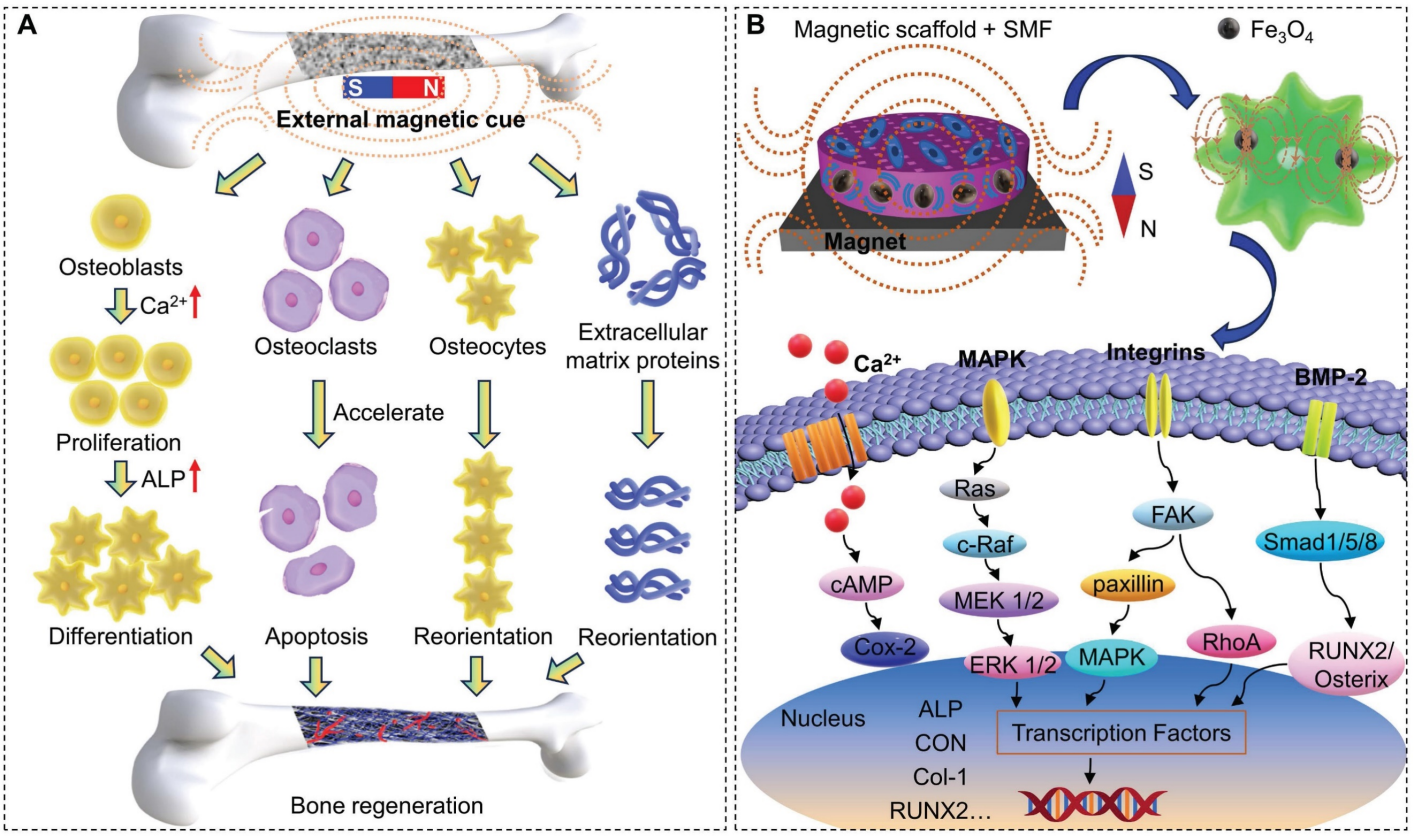
(A) Schematic of SMF promoting bone regeneration. (B) Schematic representation of magnetic scaffolds combined with SMF synergistically promoting osteogenic differentiation.

Magnetic fields transmit the magnetic force between objects around a magnet or an electric current and are mainly divided into static magnetic field (SMF) 165, electromagnetic field 166, AMF 167, and rotating magnetic field 168. Cell proliferation depends on various factors, such as the type, intensity, frequency, and duration of magnetic fields, so it is essential to determine the magnetic field range in which biological systems respond significantly 160. Among them, SMF, as a magnetic field with constant strength and fixed directions, is the subject of research in bone regeneration. SMF can be divided into weak (<1 mT, such as geomagnetic field), moderate (1 mT-1 T, such as common permanent magnets) and high (>1 T, like superconducting magnets) categories according to the magnetic field strength 169. Magnetic fields of moderate intensity are most widely used, while strong SMF of 5-10 T has also been reported to have the ability to regulate the orientation of matrix proteins and cells 170,171. As shown in Figure 6A, upon magnetic field stimulation, diamagnetic cell membranes alter membrane flux, activate intracellular ion channels, accelerate cell metabolism, and promote cell adhesion, proliferation and differentiation 172. Long-term local SMF stimulation can enhance the binding of the implant to the host tissue, prevent the loss of bone density caused by surgical invasion, promote mineralization, and accelerate bone defect healing 173. High magnetic flux inhibits osteoclast formation and differentiation by reducing tartrate-resistant acid phosphatase activity, leading to osteoclast apoptosis and necrosis 174. SMF regulates the orientation of diamagnetic osteoblasts and extracellular matrix proteins, which helps to accelerate the growth process of osteoblasts 170,171.

There are several potential physical mechanisms about SMF interacts with bone tissue 175,176: (1) Magneto-mechanical interaction. Bone is a tissue with minimal diamagnetism. In a homogeneous magnetic field with a sufficiently large magnetic gradient, material with anisotropic susceptibility rotates under magnetic torque drive until it reaches a stable orientation. In this case, phospholipid molecules will rotate and orient in the direction of the magnetic field, which expands the ion channels in the cell membrane and allows ions to pass through, increasing electrical conductivity and generating a strong current, followed by a series of biological effects that promote bone formation. (2) Electrodynamic interaction (also known as Hall effect). The COL of the bone matrix is negatively charged, and cations are adsorbed to the surface by electrostatic interactions. At the same time, there is a voltage difference between the inside and outside of the cell membrane, which is usually positively charged on the outside and negatively charged on the inside. The charged ions move between the bone matrix and the cell membrane are subjected to the Lorentzian force in the magnetic field, forming a Hall voltage to induce further ion migration and improve the permeability of the cell membrane, promoting the extracellular ions to pass through the cell membrane and enhance cell activity. (3) Radical pair effect. SMF of moderate or weak strength affects the free radical or electron spin state of some biochemical reaction intermediates, altering the rate, yield, or product distribution of the reaction. (4) Activation of the cyclic adenosine phosphate system. Magnetic stimulation can activate the cyclic adenosine monophosphate system, which in turn activates various enzyme systems to induce special physiological functions of osteocytes and accelerate bone growth. Magnetism activates multiple signaling pathways within cells that synergistically promote osteogenesis, mineralization, and angiogenesis. These pathways include:

A) Activation of the BMP-2/Smad pathway to significantly up-regulate the expression of osteogenesis-related genes and vascular endothelial growth factor 177.

B) Activation of cyclic guanosine monophosphate (cGMP)/protein kinase G (PKG)/extracellular signal-regulated kinase (ERK) 162, TGFβ-Smads 164, and YAP/β-catenin signaling pathways 178.

C) Up-regulation of the expression of bone-related genes (Runx2 and Osterix) and ALP activity. Activation of integrin signaling pathways, such as FAK, paxillin, RhoA, mitogen-activated protein kinase, and nuclear factor-kappaB, and up-regulation of BMP-2 and phosphorylation of Smad1/5/8 (Figure 6B) 24,172.

### Magnetic composite scaffolds

MNPs, such as γ-Fe_2_O_3_ and Fe_3_O_4_, have shown great potential in bone tissue engineering due to their favorable chemical stability, biocompatibility, and high magnetic torque. In particular, low-dose MNPs possess a high safety profile *in vivo* as they can be absorbed as iron ions through ionization and participate in iron homeostasis. Importantly, the US FDA approved iron oxide nanoparticles for treating iron deficiency anemia 179. In some studies, γ-Fe_2_O_3_ and Fe_3_O_4_ have been found to generate shear stress at the cellular level, promoting bone angiogenesis regardless of SMF 180,181. Iron oxide nanoparticles and SMF alone could promote osteogenic differentiation and inhibit osteoclast activity, while the combination had a stronger effect 160. In conjunction with 0.2 T AMF, α‑Fe_2_O_3_/γ‑Fe_2_O_3_ nanocomposites significantly reduced the expression of TNF-a, an osteoinductive cytokine, which induces osteoclast formation and leads to osteoporosis, increases Ca/P ratio and the expression of key bone formation markers OPN, col-1, OCN, DMP-1, and BMP-2, thereby promoting osteogenic differentiation 182. Despite the obvious advantages, the toxicity and transient residence of MNPs exposed in large quantities to bone defects limit their practical applications. The construction of magnetic composites/scaffolds helps to overcome these shortcomings and promote clinical translation.

Magnetic composite scaffolds based on γ-Fe_2_O_3_, Fe_3_O_4_, and CoFe_2_O_4_ present special magnetic field responsiveness and can be used for tissue engineering, such as orthopedic implantation and intervention 25,183,184, especially for early bone fixation and repair 185. Superparamagnetic scaffold combined with SMF demonstrates a stronger stimulating effect on cell proliferation and differentiation, and synergistically promotes the formation, integration, and remodeling of new bone 186. Under an exogenous magnetic field, PLGA microspheres encapsulated with superparamagnetic iron oxide nanoparticles can significantly promote bone repair. When the actual feeding amount of iron oxide nanoparticles was 1.38%, the microspheres were labeled as PFe-II. In the rat femoral defect experiment, after 6 weeks of treatment with PFe-II combined with magnetic field, bone mineral density (263.97 ± 25.99 mg/cm^3^), trabecular thickness (0.58 ± 0.08 mm), and bone volume/total volume (78.28 ± 5.01%) were significantly higher than those of the PFe-II group (bone mineral density, 194.34 ± 26.71 mg/cm^3^; trabecular thickness, 0.41 ± 0.07 mm; bone volume/total volume, 50.49 ± 6.41%). Moreover, the expressions of ALP, COL-I, OPN, and OCN in the repairing bone were significantly higher in the combination group than the PFe-II group, further clarifying the synergistic effect of the magnetic composites and the SMF on promoting bone regeneration 187.

**Figure 7:**
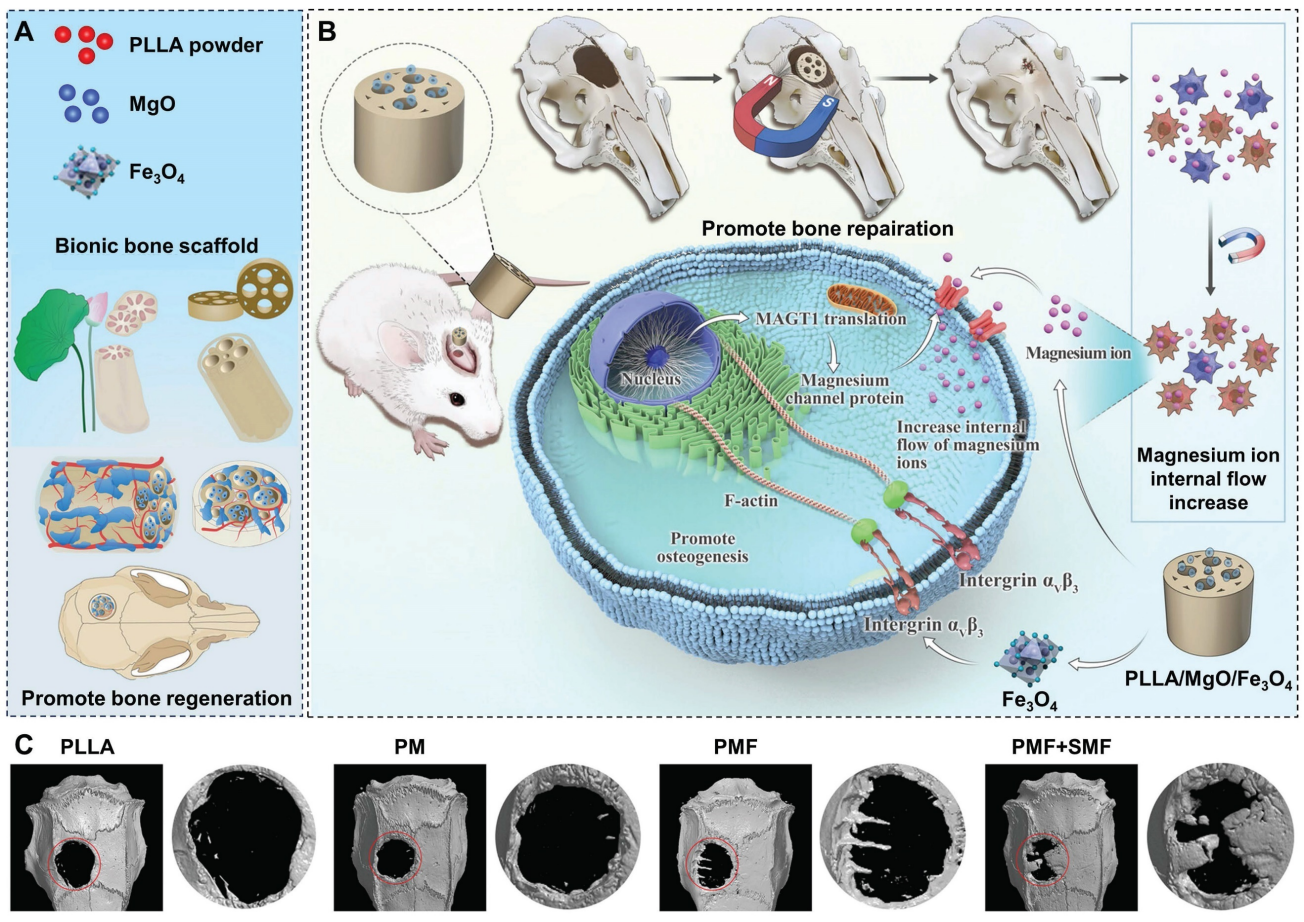
Schematics of (A) a biomimetic bone repair scaffold with a lotus seedpod-structured porous structure and (B) remote and accurate regulation of channel protein activity on the membrane of rBMSCs by SMF to promote Mg^2+^ influx and osteogenesis. (C) Micro-CT reconstruction on the horizontal plane and mid-sagittal plane of bone defect region 12 weeks after implantation, The red circle indicates the defect area. Reproduced with permission from 163. Copyright 2023, Wiley-VCH.

MgO and magnetic Fe_3_O_4_ nanoparticles were loaded into the PLLA matrix to fabricate the biomimetic porous PLLA/MgO/Fe_3_O_4_ (PMF) scaffolds via selective laser sintering (Figure 7A). With SMF, the magnetic torque effect of PLLA/MgO/Fe_3_O_4_ scaffolds (via integrin ***α***V ***β***3/actin) enhanced the activity of Mg^2+^ channel proteins, thus facilitating the capture of Mg^2+^ by BMSCs in the microenvironment and inducing osteogenesis (Figure 7B). Coordinated with SMF, magnetic scaffolds increased the expression of osteogenesis-related genes (ALP, Runx2, OCN, and OPN) and mineralization *in vitro*. After 12 weeks of scaffold implantation, 3D reconstruction images showed that PM (PLLA/MgO), PMF, and PMF+SMF groups all promoted new bone formation to a certain extent. Among them, the PMF+SMF group exhibited the most obvious trend of new bone infiltration from the edge of the scaffold to the internal pores, further confirming its ability to promote bone differentiation and regeneration (Figure 7C) 163.

Exosomes derived from BMSCs pretreated with magnetic fields or low-dose MNPs also showed enhanced osteogenic and angiogenesis effects during bone regeneration 188. Taking advantage of the responsiveness of MNPs to the SMF, dynamic stiffness of these scaffolds could be realized by changing the arrangement of magnetic particles to achieve an ideal biomimetic dynamic mechanical microenvironment and promote bone regeneration 189. Hydrogels based on superparamagnetic HA nanorod (MagHA) with continuous biophysical and biochemical gradients generated incremental HA, mechanical and electromagnetic signals under external SMF stimulation to repair osteochondral units with perfect heterogeneity 190. In addition to the dynamic structure generated by magnetic field responsiveness, MNPs could also optimize the interaction between cells and scaffolds, allowing cells to grow under natural biomimetic conditions. The magnetic scaffolds prepared by layer-by-layer assembly of superparamagnetic γ-Fe_2_O_3_ nanoparticles on the surface of PLGA/PCL electrospun scaffold could effectively promote the osteogenic differentiation of adipose-derived stem cells. Surface modification with γ-Fe_2_O_3_ improved interface hydropathy, elasticity, affinity for stem cells, and osteogenic differentiation properties, providing a novel and convenient strategy for scaffolds with active stimulation and remote control functions 191.

## Conclusions and perspectives

The ideal scaffolds to treat bone defect should provide structural support and promote specific biological activity required during bone healing. Despite growth factors are often introduced into traditional bone repairing scaffolds to stimulate new bone formation, they have many disadvantages such as severe adverse effects, short half-life and high cost. In recent years, a variety of intelligent stimulation-responsive bone-repairing materials have been reported. These materials can respond to exogenous stimuli and promote bone repair. During bone healing, multiple signaling pathways are activated to accelerate bone regeneration through biomaterials-mediated mild thermal effect, electrical and magnetic signals. With advantages of intelligent responsiveness, strong controllability, low invasiveness, and high safety, such technology provides a promising strategy for treatment of bone defect.

Besides applications in anti-tumor strategies, eliminating infection, and promoting wound healing, thermal effect also shows excellent potential in the treatment of orthopedic diseases. The mild thermal effect promotes osteocyte proliferation by enhancing metabolism at the injured site, improving expression of osteogenesis-related proteins (such as ALP and HSPs), regulating inflammatory processes, and stimulating angiogenesis. Bone repairing materials loaded with photosensitizers or paramagnetic nanomaterials generate mild thermal effects when stimulated by NIR light or magnetic field, thereby regulating endogenous signaling pathways and accelerating bone regeneration. Various biomaterials responsive to NIR light and magnetic fields have been developed and incorporated into bone repairing scaffolds, demonstrating their efficacy to promote bone regeneration. NIR laser is considered a safe and effective means of external stimulation due to its unique spatio-temporal selectivity and few side effects. NIR light triggered PTT is commonly used for superficial tissue regeneration. In contrast, the magnetic field presents better tissue penetration, and MTT is more conducive to repairing deep tissue defects. In order to avoide damage to normal tissues, it is worth noting that three-dimensional, real-time, and accurate temperature monitoring is desired to realize therapeutic effects at the most extent.

Bioelectricity is one of the common physiological signals. Implantation of electroactive biomaterials helps to restore the bone surface potential at the site of bone defects wherein endogenous bioelectricity is subnormal and promotes bone healing. Electrical stimulation generally instigates bone regeneration by activating cellular voltage-gated Ca^2+^ channels, promoting the expression of integrins and bioactive factors, increasing local blood flow, and regulating cellular signal transduction pathways. Conductive materials are capable of charge transport at the cell membrane interface and regulate cell contact with cells, tissues and biological interfaces. However, conductive materials usually need to be connected to an external power supply, which is not convenience to clinical operation. Piezoelectric materials are characterized by spontaneous electricity. Under the stimulation of mechanical stress or strain, electric polarization occurs inside the piezoelectric material, and a charge with the opposite sign is generated and accumulated on the surface, thus promoting bone structure remodeling and regeneration. Piezoelectric biomaterials often require direct contact with damaged tissue to obtain local low-level electric fields induced by body motion, but it is challenging to achieve efficient and consistent piezoelectric output. In addition, magnetoelectric materials and photoelectric materials exhibit unique capacity for tuning electrical properties with magnetic field or light modulation, and can be activated *in situ* for bone defect repair. However, the microenvironment of different bone tissues varies greatly, bringing challenges to electroactive materials for clinical translation.

Despite MNPs or magnetic signals alone can significantly promote osteogenic differentiation, their combination can further improve the efficiency of bone repair. During bone healing, magnetic signals promote bone regeneration mainly through magnetic-mechanical and electric-dynamic interactions, free radical pair effect, and activation of cyclic adenosine monophosphate. Currently, the frontier technology of magnetic-assisted bone tissue engineering mainly focuses on bone repairing scaffolds incorporated with MNPs, whereas single MNPs are rarely used. This is because the distribution of MNPs within the composite can lead to differences in magnetic moment gradients, resulting in positive impacts on cell activity, and the composite helps to prolong the residence time of the MNPs *in vivo*. Driven by an exogenous magnetic field, MNPs are always in dynamic motion. Therefore, it is critical to carry out non-invasive tracking technique and explore the degradation and distribution of MNPs during bone healing to better regulating their performance.

From the above introduction, it can be seen that for biomaterials that can generate thermal effect, electrical and magnetic signals to treat bone defect under external stimulation, conductive materials require the most complicated* in vivo* operation. When it comes to controllability, photothermal materials and conductive materials are more likely to mediate controllable physical cues in bone defect site, because photothermal materials combined with laser irradiation can bring relatively stable thermal effect, and conductive materials can also generate stable potential. The resulting temperature or potential can be conveniently regulated by adjusting the external stimulus. For piezoelectric materials, the surface potential may decrease due to electrostatic adsorption of proteins with prolonged implantation time. The arrangement of MNPs in bone defects is affected by the magnetic field. Uneven distribution of MNPs leads to inconsistent magnetic moment gradients, which affect cell behavior and bone regeneration. From the perspective of therapeutic outcomes, photothermal materials to treat deep tissue defects is less efficient than that of electroactive and magnetic materials due to the limited tissue penetration depth of NIR light. Although NIR optical fibers can be implanted into deep tissue defects to irradiate photothermal materials with improved efficacy, this invasive procedure usually causes additional damage to patients. The same dilemma also exists in conductive materials due to the requirement of metal wire to connect external electric fields. In contrast, magnetic and piezoelectric materials have advantages in implementing patient-friendly, non-/less invasive treatments.

It is still challenging to employ biomaterial-mediated *in situ* physical cues to promote osteogenesis. First, although the effectiveness of biomaterial-mediated physical cues (mild thermal effect, electrical and magnetic signals) in bone repair has been extensively demonstrated and the underlying mechanisms have been proposed, bone repair is a complex process involving multiple physiological activities, and current research is still limited. Extensive collaboration and clinical trials are needed to further investigate the exact mechanisms.

Second, biomaterials-mediated physical cues are less effective than growth factors in promoting bone regeneration. Therefore, there is an urgent need to develop intelligent bone repairing materials with better performance and greater sensitivity to external stimuli. In addition, other physical cues can also significantly promote bone repair, such as ultrasound, which can accelerate bone regeneration processes 192,193. Under pulsed ultrasound irradiation, the acoustically responsive scaffold generates enhanced acoustic trapping force to recruit stem cells, promoting bone tissue regeneration 194. Ultrasound-trigged micro/nanorobots also demonstrate potential applications in multidisciplinary fields 195. Scaffolds combining multiple stimulus-responsive bone repairing materials with improved functionalities might be a more effective strategy to treat bone defect.

Third, in existing reports, conditions, such as time, frequency, and intensity of applied external stimuli, vary significantly among different biomaterials and experimental models, making it challenging for follow-up studies. It is necessary to carry out multidisciplinary cooperation to further investigate the impacts of different stimulation parameters on the physiological microenvironment of different bone tissues.

Fourth, the strength of physical cues mediated by biomaterials depends primarily on the strength of the received external stimulus and homogeneous distribution of stimulus-response components within scaffolds. Heterogeneous output of physical cues may lead to inconsistent rates of new bone growth. Therefore, achieving efficient and consistent signal output and monitoring signal changes in realtime are desired.

Fifth, most of the literature has primarily verified the biosafety of biomaterials associated with physical cues. However, there is still a lack of systematic evaluation of the long-term physiological safety, metabolic mechanisms, and metabolites generated by degradation.

Sixth, most of the current studies on physical cues promoting bone repair are conducted in animal models. Due to the significant differences in species, the experimental results cannot be directly extrapolated to humans, especially to the elderly group with weaker vitality.

Finally, the currently widely studied synthetic bone scaffolds mainly include metallic materials, inorganic materials (bioceramics and bone cements, etc.), organic polymers (natural polymers and synthetic polymers), and composite materials. Different materials vary greatly in rigidity, porosity, hydrophilicity and degradability, so it is necessary to select appropriate single or composite materials according to the actual application scenario. In addition, the high specific surface area, porous structure, and appropriate degree of disordered nanostructures of bone repairing scaffolds also play great influences on bone regeneration. Therefore, a variety of factors need to be comprehensively considered to design scaffolds for better bone regeneration.

In summary, underlying mechanisms and the effects of physical cues (mild thermal effect and electrical and magnetic signals) elicited by various biomaterials for bone regenration with advantages of remote actuation and on-demand activation are elucidated, which show great potential in clinical translation.

## Figures and Tables

**Table 1:** Summary of typical photothermal and magnetothermal materials for bone repair.

Classification	Materials^Ref^	Exogenous stimuli (*in vitro*)	Osteogenic impact (*in vitro*)	Animal models	*In vivo* results
Photothermal metallic materials	eP@GNRs^26^	808 nm, 0.4 W cm^-2^, 15 min	Promote osteogenic differentiation (BMSCs)	Femur fracture in mice	Promote fracture healing
	pAuPds^27^	808 nm, 2 W cm^-2^, 3 min	Promote proliferation (MC3T3-E1)	Cranial defect in rat (Ø 8 mm)	Partial bridging
Photothermal metal oxides	P and Si co-doped TiO_2_^28^	808 nm, 0.25 W cm^-2^, 3 min	Promote adhesion, proliferation, and differentiation (MC3T3-E1)	Femoral defect in rat (Ø 1.6 mm)	Partial bridging
	PPCL/Nd@WH^29^	808 nm, 0.5 W cm^-2^, 5 min	Promote osteogenic differentiation (BMSCs)	Cranial defect in rat (Ø 4 mm)	Partial bridging
Photothermal metal sulfides	PCL/MoS_2_^30^	808 nm, 0.5 W cm^-2^, 40.5 ± 0.5 °C for 60 s	Promote osteogenic differentiation (BMSCs)	Tibia defect in rat (Ø 2.5 mm × 3 mm)	Bridging
	PTEB (MoS_2_)^31^	808 nm, 1.5 W cm^-2^, 41 °C for 10 min	Promote proliferation and differentiation (BMSCs)	Cranial defect in rat (Ø 5 mm)	Bridging
	D-CuS-PEG-PCL scaffold^32^	1064 nm, 1.0 W cm^-2^, 10 min	Promote osteogenic differentiation (BMSCs)	Tibia defect in rat (Ø 3 mm × 3 mm)	Partial bridging
Photothermal carbon materials	AMAD/MP hydrogel (MXene)^33^	808 nm, 1.0 W cm^-2^, 42 ± 0.5 °C for 120 s	Promote osteogenic differentiation (MC3T3-E1)	Cranial defect in rat (Ø 5 mm)	Partial bridging
	nHA/GO/CS scaffold^34^	808 nm, 1.0 W cm^-2^, 42 ± 0.5 °C for 60 s	Promote osteogenesis (human BMSCs)	Cranial defect in rat (Ø 5 mm)	Partial bridging
	CNT-alginate gel^35^	NIR apparatus, 4.0 W cm^-2^, 42 °C for 15 min	Promote mineral deposition (MG63 and DP cells)	Cranial defect in rat (Ø 8 mm)	Partial bridging
Other photothermal compounds	BMP-2@blood clot^36^	808 nm, 0.4 W cm^-2^, 10 min	Promote osteoblast proliferation and differentiation (MC3T3-E1)	Cranial defect in mice (Ø 4 mm)	Partial bridging
	GelMA/PMMA/PDA hydrogel^37^	808 nm, 0.99 W cm^-2^, 40 ± 0.5 °C for 60 s	Promote osteogenic differentiation (BMSCs)	Cranial defect in rat (Ø 5 mm)	Partial bridging
	CS/PCL/BP/PDA@Ag scaffold^38^	808 nm, 0.45 W cm^-2^, 41 ± 0.5 ℃ for 60 s	Promote osteogenic differentiation (BMSCs)	Femoral defect in rat (Ø 2.5 mm × 3 mm)	Partial bridging
	BPs@PLGA^39^	808 nm, 1.0 W cm^-2^, 40.5 ± 0.5 ℃, 60 s	Promote osteogenesis (human BMSCs)	Tibia defect in rat (Ø 2.5 mm × 3 mm)	Partial bridging
	Apt19S-PCL-BP scaffold^40^	808 nm, 0.8 W cm^-2^, 2 min	Promote osteogenic differentiation (MSCs)	Cranial defect in rat (Ø 5 mm)	Partial bridging
Magnetothermal materials	MCPC/GM/HMFNs/CPFX/Van (ferrite nanoparticles)^15^	AMF, 2.5 kW, 200 kHz, 18 A, 5 min	Promote osteogenic differentiation and mineralization (BMSCs)	Femoral defect in rat (Ø 3 mm × 3 mm)	Partial bridging
	MION-RGD/agarose (CoFe_2_O_4_@MnFe_2_O_4_)^41^	AMF, 1.35 kA m^-1^, 5 min	Promote osteogenesis and biomineralization (MC3T3-E1)	Cranial defect in rat (Ø 5 mm)	Partial bridging

**Table 2:** Summary of typical conductive and piezoelectric materials for bone repair.

Classification	Materials^Ref^	Exogenous stimuli (*in vitro*)	Osteogenic impact (*in vitro*)	Animal models	*In vivo* results
Conductive carbon materials	PCL/MWCNTs nanofibers^86^	10 μA, 5 min, three times a week	Promote angiogenesis and mineralization (UMR-106)	Cranial defect in rat (Ø 5 mm× 2.5 mm)	Partial bridging
	PCL/graphene scaffolds^87^	N/A	Promote proliferation (MC3T3 and THP-1)	Cranial defect in rat (Ø 5 mm)	Bridging
	BP-CNTpega-gel^88^	100 mV mm^-1^, 20 Hz, 2 h per day	Promote osteogenic differentiation (MC3T3)	Femoral and spinal defects in rabbit	N/A
Conductive metals	GelMA-PdMGSMW gels (Pd)^89^	4 V, 1 Hz, 10 ms for 2 continuous days	Regulate adhesion and differentiation (C2C12)	N/A	N/A
Conductive polymers	FPAT membranes (aniline trimer)^90^	N/A	Promote osteogenic differentiation (MSCs)	Cranial defect in rat (Ø 5 mm)	Partial bridging
Piezoelectric cements	K_0.5_Na_0.5_NbO_3_^91^	5 kV cm^-1^, 20 min	Promote osteogenic differentiation (BMSCs)	Femoral defect in rabbit (5 mm × 3 mm)	Partial bridging
	BaTiO_3_/Ti6Al4V scaffolds^92^	11.5 kV, 30 min	Promote osteogenic differentiation (MSCs)	Vertebral defect in sheep (12 mm × 6 mm)	Partial bridging
	PLLA/Ca/Mn co-doped BaTiO_3_^93^	6 kV cm^-1^, 30 min	Promote osteogenic differentiation (BMSCs)	Cranial defect in rat (Ø 5 mm)	Partial bridging
	BaTiO_3_/P(VDF-TrFE) membranes^94^	13 kV, 30 min	Promote osteogenic differentiation (BMSCs)	Cranial defect in rat (Ø 5 mm)	Partial bridging
	BaTiO_3_/PLA^95^	6 kV, 10 min	Promote adhesion and proliferative (MC3T3-E1)	Cranial defect in rat (Ø 5 mm)	Partial bridging
Piezoelectric polymers	MXene/PVDF membranes^96^	13 kV	Promote osteogenic differentiation (MC3T3-E1)	Cranial defect in rat (Ø 5 mm)	Partial bridging
	PVFT-BGM scaffolds^97^	13 kV, 60 °C, 60 min	Promote osteogenic differentiation (BMSCs)	Cranial defect in rat (Ø 5 mm)	Partial bridging
	P(VDF-TrFE) membranes^98^	13 kV, 60 °C, 60 min	Promote osteogenic differentiation (BMSCs)	Cranial defect in rat (Ø 5 mm)	Partial bridging
	PLLA nanofiber scaffold^99^	0.08 MPa, 20 min per day	Promote chondrogenic differentiation (ADSCs)	Femoral articular cartilage defect in rabbit (Ø 4 mm × 2 mm)	Bridging

**Table 3:** Summary of typical magnetic biomaterials for bone repair.

Materials^Ref^	Type	Intensity (*in vitro*)	Osteogenic impact (*in vitro*)	Animal models	*In vivo* results
CS/PDA@MS (Fe_3_O_4_)^161^	SMF	N/A	Promote adhesion and proliferation (BMSCs)	Humerus defect in rat (Ø 2 mm × 2 mm)	Bridging
RSF/TA/Fe_3_O_4_ hydrogel^162^	SMF	125 mT	Promote osteoblast differentiation (BMSCs)	Cranial defect in rat (Ø 5 mm)	Partial bridging
PLLA/MgO/Fe_3_O_4_ scaffolds^163^	SMF	70-80 mT	Promote osteogenesis and mineralization (BMSCs)	Cranial defect in rat (Ø 8 mm)	Partial bridging
Fe_3_O_4_/PDA coating^164^	SMF	15 mT	Promote proliferation and osteoblast differentiation (human BMSCs)	Femoral defect in rabbit (Ø 5 mm × 10 mm)	Partial bridging

## Abbreviations

NIR: Near-infrared
AMF: Alternating magnetic field
MNPs: Magnetic nanoparticles
MSCs: Mesenchymal stem cells
HSPs: Heat shock proteins
ALP: Alkaline phosphatase
PTT: Photothermal therapy
BP: Black phosphorus
PDA: Polydopamine
BMSCs: Bone mesenchymal stem cells
PCL: Poly(ε-caprolactone)
CDs: Carbon dots
GO: Graphene oxide
CNTs: Carbon nanotubes
HA: Hydroxyapatite
CS: Chitosan
ROS: Reactive oxygen species
PLGA: Poly(lactic-*co*-glycolic acid)
PCMs: Phase change materials
MTT: Magnetothermal therapy
PMMA: Polymethyl methacrylate
RGD: Arg-Gly-Asp
COL: Collagen
TGF-β: Transforming growth factor-β
BMP: Bone morphogenetic protein
FAK: Focal adhesion kinase
PLA: Poly-DL-lactide
Ppy: Polypyrrole
PANI: Polyaniline
FPAT: Polyurethane
PVDF: Polyvinylidene fluoride
P(VDF-TrFE): Poly(vinylidene fluoridetrifluoroethylene)
β-TCP: β-Tricalcium phosphate
PHB: Poly[(*R*)3-hydroxybutyrate]
PHBV: Poly[3-hydroxybutyrate-*co*-3-hydroxyvalerate]
SMF: Static magnetic field
cGMP: Cyclic guanosine monophosphate
PKG: Protein kinase G
ERK: Extracellular signal-regulated kinase
